# Prognostic significance of *BRAF V600E* and *TERT* promoter mutations in radioiodine resistance and recurrence of differentiated thyroid cancer

**DOI:** 10.1097/MD.0000000000044540

**Published:** 2025-09-19

**Authors:** Le Thi Tuyet Nhung, Nghiem Xuan Hoan, Dao Phuong Giang, Dang Trung Dung, Nguyen Thi Phuong, Ngo Thi Minh Hanh, Le Van Khanh, Ngo Thu Hang, Le Ngoc Ha, Hoang Van Tong

**Affiliations:** a 103 Military Hospital, Vietnam Military Medical University, Hanoi, Vietnam; b Cancer Center, 19-8 Hospital, Hanoi, Vietnam; c Vietnamese-German Center for Medical Research (VG-CARE), 108 Military Central Hospital, Hanoi, Vietnam; d Department of Thoracic Surgery, 108 Military Central Hospital, Hanoi, Vietnam; e Department of Nuclear Medicine, 108 Military Central Hospital, Hanoi, Vietnam; f Department of Pathology, 108 Military Central Hospital, Hanoi, Vietnam; g Institute of Biomedicine and Pharmacy, Vietnam Military Medical University, Hanoi, Vietnam; h Department of Pathophysiology, Vietnam Military Medical University, Hanoi, Vietnam.

**Keywords:** ¹³¹I treatment, *BRAF V600E* mutation, differentiated thyroid cancer, radioactive iodine therapy, *TERT* promoter mutation

## Abstract

Thyroid cancer is the most common endocrine malignancy, with rising resistance to radioactive iodine therapy in differentiated thyroid cancer (DTC). *BRAF V600E* and telomerase reverse transcriptase (*TERT*) promoter mutations synergistically drive recurrence and mortality in papillary thyroid cancer. This study investigates their association with radioiodine avidity loss, clinical features, and prognostic significance in Vietnamese patients with radioiodine-refractory differentiated thyroid cancer (RAIR-DTC). We analyzed the diagnostic and prognostic values of *BRAF V600E* and *TERT* promoter mutations in 144 patients with DTC who underwent total thyroidectomy and received ¹³¹I treatment from January 2021 to December 2024. The *BRAF V600E* mutation was detected in 81.9% (118/144) of cases, while the *TERT* promoter mutation was found in 38.2% (55/144). *TERT* mutations were more frequent in RAIR-DTC patients than in radioiodine-responsive cases, increasing the risk of radioiodine avidity loss (OR = 3.6, *P* = .007), while *BRAF V600E* alone showed no significant impact. Coexisting *BRAF V600E* and *TERT* mutations independently elevated the risk of loss of radioiodine avidity (OR = 4.8, *P* = .009). Both mutations were linked to tumor size, thyroglobulin levels, recurrence/metastasis, and higher recurrence risk, with co-mutation accelerating recurrence. *TERT* mutations distinguished RAIR-DTC from responsive cases (AUC = 0.63) and moderately predicted recurrence (AUC = 0.72), similar to co-mutation (AUC = 0.69). Postoperative recurrence-free survival was shorter in co-mutated cases (10.5 months) than in *BRAF*-negative (11.3 months) or *BRAF*-only (11.8 months) groups (*P* < .05). Our study suggests that *TERT* promoter mutations contribute to an increased risk of radioactive iodine treatment failure in patients with DTC. These mutations may serve as an early biomarker for radioiodine resistance.

## 1. Introduction

Thyroid cancer is the most common malignant endocrine disorder, with 821,214 cases and 47,507 deaths reported worldwide in 2022.^[1]^ Differentiated thyroid cancer (DTC) is the most prevalent type, accounting for over 92% of thyroid cancer cases.^[2]^ DTC originates from follicular thyroid cells and includes papillary thyroid carcinoma (PTC), follicular thyroid carcinoma, and Hürthle cell carcinoma. In Vietnam, thyroid cancer ranks sixth in cancer incidence, with a rate of 4.8 per 100,000 people.^[1]^ The primary treatment methods for DTC include surgery, radioactive iodine (RAI) therapy, and thyroid-stimulating hormone (TSH) suppression therapy. These treatments have been shown to be effective, with a 5-year survival rate of approximately 83% to 98%.^[3]^ RAI is particularly effective in reducing recurrence and mortality in DTC patients. However, approximately 5% to 15% of DTC patients are resistant to ¹³¹I treatment, with an average survival time of 2.5 to 3.5 years.^[4]^

The ability of DTC cells to uptake iodine plays a crucial role in the effectiveness of radioactive iodine treatment. Poor iodine uptake by thyroid cancer cells is largely due to the inactivation of the sodium iodide symporter (NIS) protein, resulting in the loss of its function and the cells’ inability to capture and absorb iodine. This is considered the primary mechanism behind resistance to ¹³¹I treatment. Recent studies have focused on mutations in specific genes that play key roles in the pathogenesis of thyroid cancer.^[5,6]^ Molecular biomarkers, such as *BRAF V600E*, *RAS*, RET/PTC, and PAX8/PPARγ chromosomal rearrangements, as well as phosphatidylinositol 4,5-bisphosphate 3-kinase catalytic subunit α, tumor protein p53, RAC-α serine/threonine-protein kinase 1, and telomerase reverse transcriptase (*TERT*), play a crucial role in the diagnosis, treatment, and monitoring of advanced thyroid cancer.^[7]^

The *BRAF V600E* mutation is associated with reduced radioiodine avidity and failure of radioiodine treatment, particularly in PTC patients.^[8,9]^ The *BRAF V600E* mutation causes constant activation of the MAPK/ERK (mitogen-activated protein kinase/extracellular signal-regulated kinase) signaling pathway, leading to excessive cell division. The MAPK/ERK pathway typically regulates cell division, differentiation, and apoptosis in normal cells, but in cells with the *BRAF V600E* mutation, it becomes hyperactive, driving uncontrolled cell proliferation and tumor formation. Studies have shown a correlation between the *BRAF V600E* mutation and adverse histopathological features of PTC, including invasiveness, loss of radioactive iodine uptake, and treatment failure.^[10]^ Activation of the MAPK pathway through the *BRAF V600E* mutation also leads to the release of thrombospondin 1 into specific extracellular matrix microenvironments, where it interacts with and modulates proteins such as integrins, non-integrin cell membrane receptors, matrix proteins, cytokines, vascular endothelial growth factor A, and matrix metalloproteinases. This interaction stimulates thyroid cancer cell signaling pathways, further promoting tumor progression and metastasis.^[11]^

*TERT* is the catalytic subunit of telomerase, an enzyme responsible for maintaining chromosomal integrity and genome stability.^[12,13]^ In normal cells, telomeres shorten with each division, eventually leading to cellular senescence. However, the reactivation of telomerase prevents telomere shortening, contributing to the immortality of cancer cells. *TERT* plays a crucial role in telomerase activation during malignant cell transformation and in the regulation of genes involved in cell growth.^[14,15]^ Studies also suggest that *TERT* can function as an oncogene, with *TERT* promoter mutations potentially playing a significant role in thyroid cancer.^[16,17]^

The combination of *BRAF V600E* and *TERT* promoter mutations has a strong synergistic effect on recurrence and mortality rates in PTC.^[6]^ A recent study revealed that the coexistence of *BRAF V600E* and *TERT* promoter mutations is strongly associated with radioiodine resistance and impaired iodine metabolism in recurrent PTC.^[18]^ This dual mutation may serve as a biomarker for radioiodine treatment failure in PTC. Therefore, in this study, we aim to investigate the association between *BRAF V600E* and *TERT* mutations, the loss of radioiodine avidity, and the clinical characteristics as well as their diagnostic and prognostic value in Vietnamese patients with radioiodine-refractory differentiated thyroid cancer (RAIR-DTC).

## 2. Materials and methods

### 2.1. Ethical statement

The treatment and study protocols followed the institutional instructions. Written informed consent was obtained from all study participants. The study was approved by the Military Hospital 103, Vietnam Military Medical University (Ethics approval nr.: 734/CNChT-HĐĐĐ).

### 2.2. Study subjects

We enrolled 144 patients with DTC who underwent total thyroidectomy and had residual, recurrent, or metastatic lesions following surgery, subsequently receiving ¹³¹I treatment from January 2021 to December 2024. These patients were diagnosed based on histopathology according to the World Health Organization 2022 classification of thyroid cancer.^[19]^ Residual, recurrent, and metastatic lesions were identified through histopathological examination of lymph node specimens or other lesions after surgery. Tissue samples from residual, recurrent, or metastatic sites were stained with hematoxylin and eosin, and the results were analyzed using optical microscopy, with histopathological classification following the World Health Organization 2022 guidelines.^[19]^ Baseline and clinical characteristics of patients, including age, sex, disease stage (according to the American Joint Committee on Cancer 8th edition),^[20]^ recurrence risk stratification (according to American Thyroid Association [ATA] 2015),^[21]^ history of ^131^I treatment, and serum thyroglobulin (Tg) levels before the first ¹³¹I treatment, were recorded.

The patients were divided into two groups based on their resistance to ¹³¹I treatment. The first group consisted of patients with RAIR-DTC (n = 111), while the second group included patients with radioiodine-responsive differentiated thyroid cancer (RR-DTC) (n = 33). RAIR-DTC was defined according to one of the 4 criteria outlined in the 2015 American Thyroid Association (ATA) management guidelines.^[21]^ RR-DTC included patients who did not meet any of the four criteria for radioiodine resistance, as per the 2015 ATA guidelines. Each patient was followed up for at least 12 months after surgery using a comprehensive set of criteria, including clinical features, serum Tg testing, and diagnostic imaging (whole-body scan, CT, PET/CT, etc.). Exclusion criteria included patients with non-DTC, those with other types of cancer, and patients who did not consent to participate in the study. The baseline characteristics of the patients are presented in Table 1.

**Table 1:** Clinicopathologic characteristics of the study groups.

Characteristics	DTC (n = 144)	RAIR-DTC ^131^I (n = 111)	RR-DTC ^131^I (n = 33)	*P*-value
*Age (years*)				
Mean (±SD)	43.8 ± 13.9	44.9 ± 13.8	40.2 ± 14.1	>.05†
<55 (%)	107 (74.3)	81 (73)	26 (78.8)	>.05*
≥55 (%)	37 (25.7)	30 (27)	7 (21.2)	
*Gender*				
Female	107 (74.3)	83 (74.8)	24 (72.7)	>.05*
Male	37 (25.7)	28 (25.2)	9 (27.3)	
*Tumor size (T*)				
T1–T2	96 (66.7)	78 (70.3)	18 (54.5)	**<.05** *
T3–T4	41 (28.5)	26 (23.4)	15 (45.5)	
Tx	7 (4.9)	7 (6.3)	0 (0)	
*Lymph node metastasis (N*)				
N0	31 (21.5)	24 (21.6)	7 (21.2)	>.05‡
N1a	26 (18.1)	19 (17.1)	7 (21.2)	
N1b	86 (59.7)	67 (60.4)	19 (57.6)	
Nx	1 (0.7)	1 (0.9)	0 (0)	
*Distant metastasis (M*)				
M0	144 (100)	111 (100)	33 (100)	N/A
M1	0 (0)	0 (0)	0 (0)	
*TNM staging system*				
I	113 (78.5)	86 (77.5)	27 (81.8)	>.05‡
II	25 (17.4)	20 (18)	5 (15.2)	
III	5 (3.5)	4 (3.6)	1 (3)	
IV	1 (0.7)	1 (0.9)	0 (0)	
*Risk of recurrence*				
Low	5 (3.5)	3 (2.7)	2 (6.1)	>.05‡
Medium	37 (25.7)	29 (22.1)	8 (24.2)	
High	102 (70.8)	79 (71.2)	23 (69.7)	
*RAI total dose (mCi*)				
<200	44 (30.6)	18 (16.2)	26 (78.8)	**<.001** ‡
200–399	72 (50)	67 (60.4)	5 (15.2)	
400–599	17 (11.8)	16 (14.4)	1 (3)	
≥600	11 (7.6)	10 (9)	1 (3)	
Total dose (medium)	252.5 (150–350)	280 (225–375)	150 (125–162.5)	**<.001** §
*The number of* ^*131*^*I treatments*				
1	41 (28.5)	16 (14.4)	25 (75.8)	**<.001** *
2	63 (43.8)	57 (51.4)	6 (18.2)	
3	22 (15.3)	21 (18.9)	1 (3)	
4	10 (6.9)	9 (8.1)	1 (3)	
≥5	8 (5.6)	8 (7.2)	0 (0)	
*Time of recurrence and metastasis*				
<1	40 (27.8)	20 (18)	20 (60.6)	**<.001** ‡
1–3	57 (39.6)	46 (41.4)	11	
3–5	20 (13.9)	19 (17.1)	1 (3)	
>5	27 (18.8)	26 (23.4)	1 (3)	
Medium	19.5 (9–45.5)	29 (15–55)	8 (6–15)	**<.001** §
*Characteristics of recurrence and metastasis*				
Lymph node recurrence and Lung metastasis	7 (4.9)	5 (4.5)	2 (6.1)	>.05‡
Lymph node recurrence and Lung and bone metastases	2 (1.4)	2 (1.8)	0 (0)	
Only lymph node recurrence	135 (93.8)	104 (93.7)	31 (93.9)	
*Neck recurrence and regional metastasis*				
Central neck lymph nodes	19 (13.2)	16 (14.4)	3 (9.1)	>.05*
Lateral neck lymph nodes	73 (50.7)	53 (47.7)	20 (60.6)	
Thyroid bed and lateral neck lymph nodes	1 (0.7)	1 (0.9)	0 (0)	
Central and lateral neck lymph nodes	47 (32.6)	39 (35.1)	8 (24.2)	
Thyroid bed	4 (2.8)	2 (1.8)	2 (6.1)	
Largest lymph node size (mm)	15 (10–20)	15 (10–20)	15 (10–20)	>.05§
*Histopathology of recurrent lymph nodes*				
C-PTC	120 (83.3)	90 (81.1)	30 (90.9)	N/A
DSV-PTC	2 (1.4)	1 (0.9)	1 (3)	N/A
FV-PTC	9 (6.3)	9 (8.1)	0 (0)	N/A
ccPTC	3 (2.1)	3 (2.7)	0 (0)	N/A
TCV-PTC	4 (2.8)	3 (2.7)	1 (3)	N/A
CCV-PTC	1 (0.7)	1 (0.9)	0 (0)	N/A
OV-PTC	3 (2.1)	3 (2.7)	0 (0)	N/A
HV-PTC	1 (0.7)	1 (0.9)	0 (0)	N/A
PDTC	1 (0.7)	0 (0)	1 (3)	N/A

Bold values indicate statistical significance.

ccPTC = clear cell variant of PTC; CCV-PTC = columnar cell variant of PTC; C-PTC = conventional papillary thyroid carcinoma; DSV-PTC = diffuse sclerosing variant of PTC; FV-PTC = follicular variant of PTC; HV-PTC = the hobnail variant of PTC; OV-PTC = oncocytic variant of PTC; PDTC = poorly differentiated thyroid carcinoma; PTC = papillary thyroid cancer; RAIR-DTC = radioiodine-refractory differentiated thyroid cancer; RR-DTC = radioiodine-responsive differentiated thyroid cancer; TCV-PTC = tall-cell variant of PTC; TG = thyroglobulin.

* Chi-square test.

† Student *t* test.

‡ Fisher exact test.

§ Mann–Whitney *U* test.

### 2.3. DNA extraction

DNA was isolated from FFPE tissue using the GeneJET FFPE DNA Purification Kit (Thermo Fisher Scientific, Waltham), following the manufacturer’s instructions. The DNA was eluted in TAE buffer and stored at −20 °C for short-term use and at −80 °C for long-term storage. DNA concentration and purity were assessed using a NanoDrop spectrophotometer (Thermo Fisher Scientific).

### 2.4. Real-time PCR assay for the detection of *BRAF* and *TERT* mutations

The *BRAF V600E* mutation was detected in the samples using real-time PCR following the hospital’s routine procedure. Briefly, the reaction mixture included 1X master mix (Qiagen, Hilden, Germany), 10 pM of forward and reverse primers, 5 pM of the *BRAF V600E* Mu probe, 10 pM of peptide nucleic acid (PNA) *BRAF* as an internal control, and 20 to 50 ng of genomic DNA in a total volume of 20 μL. Real-time PCR was performed under the following cycling conditions: 95 °C for 15 minutes (enzyme activation), followed by 45 cycles of 95 °C for 15 seconds (denaturation) and 60 °C for 1 minute (annealing/extension). All amplifications were carried out using the CFX96 detection system (Bio-Rad, Hercules).

Mutations in the *TERT* promoter (C228T and C250T) result in the same sequence, so a single probe was used to detect both mutations. A second probe was employed to recognize the C228 wild-type locus. The sequences of the probes used are as follows: *TERT* promoter mutant (5′-FAM/CCC+C+T+T+CCGG/3IABkFQ/) and *TERT* promoter wild-type (5′-HEX/CCC+C+C+T+CCGG/3IABkFQ/). The probes were synthesized by Integrated DNA Technologies. All reactions were performed in duplicate using 400 nM of each primer (5′-CCT GCC CCT TCA CCT TCC AG-3′ and 5′-AGA GCG GAA AGG AAG GGG A-3′). For the PNA blocker, *TERT* PNA was designed based on the *TERT C228T* sequence (NH2-CCG GAG GGG GCT-CONH2) and custom synthesized by Panagene Inc. (Daejeon, Korea). The reaction mixture also included 1x Q-Solution (Qiagen, Hilden, Germany) and 20 to 50 ng of genomic DNA in a total volume of 20 μL. Real-time PCR was conducted under the following cycling conditions: 95 °C for 15 minutes (enzyme activation), followed by 50 cycles of 94 °C for 30 seconds (denaturation) and 64 °C for 1 minute (annealing/extension), and a final stage of 98 °C for 10 minutes (enzyme deactivation). All amplifications were performed using the QuantiTect Probe RT-PCR Kit and the CFX 96 detection system (Bio-Rad, Hercules) and Agilent system (Agilent Technologies, Santa Clara).

### 2.5. Sanger sequencing for the detection of *BRAF* and *TERT* mutations

Detection of the *C228T* and *C250T TERT* promoter mutations was performed using routine PCR followed by direct sequencing with *TERT* seqF (5′-CAGCGCTGCCTGAAACTC-3′) and *TERT* seqR (5′-CGTCCTGCCCCTTCAC-3′). However, the *TERT* promoter region surrounding the mutations is characterized by a high GC nucleotide content (over 80%), which can affect PCR amplification efficiency. To optimize the conditions for challenging Sanger sequencing, the Q-Solution Kit was used. Amplification was carried out with 50 ng of gDNA input, a mixture of *TERT* seqF and *TERT* seqR primers (200 nM final concentration), and GoTaq Green Master Mix (Promega, Madison), in a 25 μL reaction. The PCR conditions were as follows: 95 °C for 5 minutes; 38 cycles of 95 °C for 30 seconds, 60 °C for 30 seconds, and 72 °C for 30 seconds; followed by 72 °C for 10 minutes. PCR products were purified using USB ExoSapit (GE Healthcare, Uppsala, Sweden) and cycle sequenced using the BigDye Terminator version 3.1 cycle sequencing kit (Applied Biosystems, Waltham) according to the manufacturer’s protocol.

### 2.6. Statistical analysis

All statistical analyses were conducted using IBM SPSS Statistics Version 22 (IBM Corp., Armonk). Categorical data were presented as frequencies and percentages. Differences between categorical variables were tested using the Chi-square test and Fisher exact test, where appropriate. Associations between *BRAF* and *TERT* mutations with radioiodine-refractory DTC or clinical parameters were evaluated using logistic regression. Variables associated with loss of radioiodine avidity, gene mutations, and second recurrence in RAIR-DTC patients, identified through univariate analysis or considered clinically relevant, were included in the multivariate logistic regression models. Receiver operating characteristic curve analysis was performed to evaluate the prognostic value of *BRAF V600E* and *TERT* promoter mutations in distinguishing between radioiodine-refractory and radioiodine-responsive DTC, as well as between recurrence and non-recurrence in RAIR-DTC patients following surgery. Progression-free survival after surgery was estimated using the Kaplan–Meier method. A *P*-value of <.05 was considered statistically significant.

## 3. Results

### 3.1. Clinicopathologic characteristics of patients with DTC

The clinical characteristics of patients with DTC are summarized in Table 1. In the group of patients with RAIR-DTC, the mean age at diagnosis was 44.9 years higher than patients with RR-DTC (40.2 years). The female-to-male ratio in both groups was approximately 3:1. We observed a significant difference in tumor size between patients with RAIR-DTC and those with RR-DTC (*P* < .05). The majority of patients had lymph node metastases (77.8%) but had not yet developed distant metastases. According to the AJCC staging system, 95.9% of patients were classified as stage I or II, while 4.2% were in stage III or IV. The number of ^131^I treatments (>3 times) and RAI total dose (mCi) were significantly higher in the RAIR-DTC group compared RR-DTC group (*P* < .001). In the RAIR-DTC group, the conventional papillary thyroid carcinoma was the most prevalent (81.1%), followed by the follicular variant (8.1%), tall-cell variant (2.7%), clear cell variant (2.7%), oncocytic variant (2.7%), diffuse sclerosing variant (0.9%), columnar cell variant (0.9%), and hobnail variant (0.9%). In contrast, in the RR-DTC group, the majority of cases were classified as the classic PTC variant (90.9%), with only 2 other variants observed: the diffuse sclerosing variant (3%) and the tall cell variant (3%). Interestingly, time of recurrence and metastasis was significantly shorter in the RR-DTC group (8 months) compared to the RAIR-DTC group (29 months) (*P* < .001) (Table 1).

### 3.2. Association between *BRAF V600E*, *TERT* promoter mutations, and loss of radioiodine avidity in patients with DTC

The *BRAF V600E* mutation was observed in 118/144 (81.9%) cases while the *TERT* promoter mutation was found in 55/144 (38.2%) cases. The frequency of *BRAF V600E* mutation was not significantly different between patients with RAIR-DTC ^131^I and those with RR-DTC ^131^I. We observed that the frequency of *TERT* promoter mutation in patients with RAIR-DTC ^131^I was significantly higher compared to those in patients with RR-DTC ^131^I. This result indicates that *TERT* promoter mutation was associated with an increased risk of loss of radioiodine avidity in patients with DTC (OR = 3.6; 95% CI: 1.4–9.3; *P* = .007). The coexistence of *TERT* promoter and *BRAF V600E* mutations was detected in 34% (49/144) of DTC patients and was significantly higher in patients with RAIR-DTC ^131^I compared to those with RR-DTC ^131^I indicating that the coexistence of *TERT* promoter and *BRAF V600E* mutations were also related to an increased risk of loss of radioiodine avidity in patients with DTC (OR = 4.8; 95% CI: 1.2–19.9; *P* = .009) (Table 2). In a multivariate analysis, we also found that the frequency of these coexisting mutations was independently associated with an increased risk of loss of radioiodine avidity in patients with DTC (OR = 4.1; 95% CI: 1.3–12.8; *P* = .013) (Table 3). However, there was no statistically significant association between age, T4 tumors, lymph node metastasis (N1), conventional papillary thyroid carcinoma, and loss of radioiodine avidity in DTC patients (*P* > .05) (Table 3).

**Table 2:** Association between *BRAF V600E*, *TERT* mutations, and loss of radioiodine avidity in DTC patients.

Mutations		DTC patient (n = 144)	RAIR-DTC ^131^I (n = 111)	RR-DTC ^131^I (n = 33)	OR (95% CI)	*P*-value
*BRAF V600E*	Negative	26 (18.1)	18 (16.2)	8 (24.2)	–	Ref
	Positive	118 (81.9)	93 (83.8)	25 (75.8)	1.7 (0.6–4.2)	.3
*TERT*	Negative	89 (61.8)	62 (55.9)	27 (81.8)	–	Ref
	Positive	55 (38.2)	49 (44.1)	6 (18.2)	**3.6 (1.3–11.3**)	**.007**
No mutation		20 (13.9)	12 (10.8)	8 (24.2)	–	Ref
*BRAF V600E* alone		69 (47.9)	50 (45)	19 (57.6)	1.8 (0.6–5.5)	.29
*TERT* mutation alone		6 (4.2)	6 (5.4)	0 (0)	NA	NA
*BRAF V600E* and *TERT* mutations		49 (34)	43 (38.7)	6 (18.2)	**4.8 (1.2–19.9**)	**.009**

*P* values were calculated by using Chi-square test. Bold values indicate statistical significance.

NA = not applicable, RAIR-DTC = radioiodine-refractory differentiated thyroid cancer, Ref = reference, RR-DTC = radioiodine-responsive differentiated thyroid cancer, *TERT* = telomerase reverse transcriptase.

**Table 3:** Univariate and multivariate analysis of risk factors for loss of radioiodine avidity in DTC patients.

Characteristics	Univariate analysis			Multivariate analysis		
	OR	95% CI	*P*-value	OR	95% CI	*P*-value
Age ≥ 55	1.4	0.5–3.5	>.05	1.1	0.4–2.7	>.05
Male	0.9	0.4–2.2	>.05	1	0.4–2.4	>.05
Tumor size T4	0.6	0.2–1.9	>.05	0.4	0.1–1.2	>.05
Lymph node metastasis N1	0.9	0.4–2.4	>.05	1.1	0.4–3.0	>.05
C-PTC	0.4	0.1–1.5	>.05	0.4	0.1–1.4	>.05
*BRAF V600E* and *TERT* mutations	**2.8**	**1.1–7.5**	**<.05**	**4.1**	**1.3–12.8**	**.013**

Bold values indicate statistical significance.

C-PTC = conventional papillary thyroid carcinoma, *TERT* = telomerase reverse transcriptase.

### 3.3. Association between *BRAF V600E*, *TERT* promoter, and clinical characteristics

Our results showed that the mean age at diagnosis, tumor size, stimulated Tg levels, and suppressed Tg levels at recurrence/metastasis in patients with RAIR-DTC carrying both *BRAF V600E* and *TERT* promoter mutations were significantly higher compared to those in the *BRAF*-negative and *BRAF*-positive only groups (*P* < .05) (Table 4). The prevalence of coexistence of *TERT* promoter and *BRAF V600E* mutations was 4.72 times higher in patients aged ≥ 55 years compared to those aged < 55 years (OR = 4.7; 95% CI: 1.5–14.7; *P* = .006). Similarly, patients with T4 tumors were 11.2 times more likely to carry both mutations compared to those with T1, T2 or T3 tumors (OR = 11.2; 95% CI: 2.1–59.5; *P* = .005). Furthermore, the prevalence of *BRAF V600E* and *TERT* promoter co-mutations was 3.3 times higher in patients with a high risk of recurrence compared to those with low-to-moderate recurrence risk (OR = 3.3; 95% CI: 1–11.1; *P* = .05) (Table 5).

**Table 4:** Association between gene mutations and clinical features in RAIR-DTC patients.

Characteristics	Mutation			*P* value
	*BRAF V600E* negative (n = 18)	*BRAF V600E* positive (n = 50)	*BRAF V600E* and *TERT* mutations (n = 43)	
*Age (years*)				
Mean	44.2 ± 11.2	40.6 ± 13.3	50.1 ± 13.8	<.005
<55	14 (77.8)	41 (82)	26 (60.5)	>.05
≥55	4 (22,2)	9 (18)	17 (39.5)	
*Gender*				
Female	11 (61.1)	41 (82)	31 (72.1)	>.05
Male	7 (38.9)	9 (18)	12 (27.9)	
*Tumor size (T*)				
T1–2	13 (72.2)	43 (86)	22 (51.2)	<.001
T3–4	2 (11.1)	5 (10)	19 (44.2)	
Tx	3 (16.7)	2 (4)	2 (4.7)	
*Lympho note metastasis (N*)				
N0	4 (22.2)	11 (22)	9 (20.9)	>.05
N1	14 (77.8)	38 (76)	34 (79.1)	
Nx	0 (0)	1 (2)	0 (0)	
*Distant metastasis (M*)				
M0	18 (100)	50 (100)	43 (100)	>.05
M1	0 (0)	0 (0)	0 (0)	
*RAI total dose (mCi*)				
Medium	252.5 (218.8–318.8)	275 (195–350)	300 (250–450)	>.05
<600	16 (88.9)	47 (94)	38 (88.4)	>.05
≥600	2 (11.1)	3 (6)	5 (11.6)	
*The number of* ^*131*^*I treatments*				
<3	14 (77.8)	35 (70)	24 (55.8)	>.05
3–5	3 (16.7)	13 (26)	19 (44.2)	
≥6	1 (5.6)	2 (4)	0 (0)	
*Suppressed thyroglobulin*				
Medium	0.12 (0.1–1.2)	1.26 (0.3–3.1)	2.46 (0.5–7.9)	<.05
<1 ng/mL	14 (82.4)	36 (76.6)	25 (62.5)	>.05
≥1 ng/mL	3 (17.6)	11 (23.4)	15 (37.5)	
*Stimulated thyroglobulin*				
Medium	7.94 (0.4–36)	27.79 (4.8–74.1)	38.2 (14.4–131.5)	<.05
<10 ng/mL	10 (55.6)	15 (31.3)	8 (19.5)	<.05
≥10 ng/mL	8 (44.4)	33 (68.8)	33 (80.5)	
*Histopathology*				
C-PTC	15 (83.3)	38 (76)	37 (86)	>.05
Other histological variants	3 (16.7)	12 (24)	6 (14)	

C-PTC = conventional papillary thyroid carcinoma, RAIR-DTC = radioiodine-refractory differentiated thyroid cancer, *TERT* = telomerase reverse transcriptase.

**Table 5:** Univariate and multivariate analysis of risk factors for *BRAF V600E*, *TERT* mutations in RAIR-DTC patients.

Characteristics	Univariate analysis		Multivariate analysis	
	OR (CI 95%)	*P*-value	OR (CI 95%)	*P*-value
Age ≥ 55	2.8 (1.2–6.5)	**<.05**	**4.7 (1.5–14.4**)	**.006**
Tumor size T4	12.8 (2.7–60.6)	**<.005**	**11.2 (2.1–59.5**)	**.005**
High risk of recurrence	3.817 (1.4–10.3)	**<.01**	**3.3 (1–11.1**)	**.05**
Number of ¹³¹I treatment ≥ 3	2.0 (0.9–4.6)	>.05	1.8 (0.6–5.1)	>.05
RAI total dose ≥ 600 mCi	1.7 (0.4–6.1)	>.05	1.7 (0.3–8.3)	>.05
Loss of iodine uptake on whole-body scan	1.3 (0.6–2.8)	>.05	1.6 (0.6–4)	>.05
Incomplete response after salvage surgery	2.4 (1.0–6.0)	>.05	2.1 (0.7–6.2)	>.05

Bold values indicate statistical significance.

RAIR-DTC = radioiodine-refractory differentiated thyroid cancer.

We also conducted univariate and multivariate analyses and found that tumor size and the presence of *BRAF V600E* and *TERT* promoter co-mutations were significant predictors of second recurrence (Table 6). Furthermore, the presence of *BRAF V600E* and *TERT* promoter co-mutations increased the risk of recurrence by 10.4-fold compared to patients without these co-mutations (OR = 10.4; 95% CI: 2.4–44.5; *P* = .002). These findings suggest that the coexistence of *BRAF V600E* and *TERT* promoter mutations significantly increases the risk of earlier recurrence in patients with RAIR-DTC.

**Table 6:** Univariate and multivariate analysis of clinical factors and mutations associated with second recurrence in RAIR-DTC patients.

Characteristics	Univariate analysis			Multivariate analysis		
	OR	95% CI	*P*-value	OR	95% CI	*P*-value
Age ≥ 55	1.2	0.4–3.5	>.05	0.8	0.2–3	>.05
Male	4.1	1.5–11.2	<.01	4.4	**1.3–15.2**	**.02**
Tumor size T4	3.0	0.9–10.3	>.05	1.2	0.3–5.1	>.05
Lympho note metastasis N1	1.2	0.4–4.0	>.05	1.6	0.4–7.4	>.05
C-PTC	0.9	0.3–3.1	>.05	0.9	0.2–4.4	>.05
Surgical treatment	**15.9**	**1.6–161.9**	**<.05**	**59.2**	**3.8–928.4**	**.004**
*BRAF*+ and *TERT*+	**5.0**	**1.7–14.3**	**<.005**	**10.4**	**2.5–44.5**	**.002**

Bold values indicate statistical significance.

C-PTC = conventional papillary thyroid carcinoma, RAIR-DTC = radioiodine-refractory differentiated thyroid cancer, TERT = telomerase reverse transcriptase.

### 3.4. Diagnostic and prognostic values of *BRAF V600E* in combination with *TERT* promoter in patients with DTC

**Figure 1.**
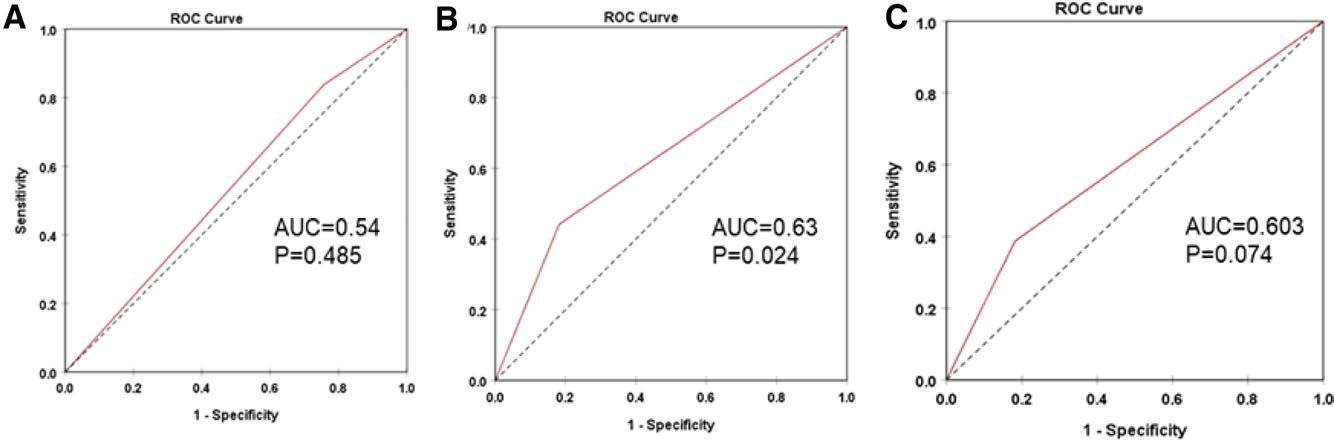
Prognostic performance of the *BRAF V600E* and *TERT* promoter mutations for loss of radioiodine avidity in patients with DTC. (A, B and C) ROC curve of *BRAF V600E*, *TERT* promoter mutations and *BRAF V600E* and *TERT* promoter co-mutation, respectively, in differentiating radioiodine-refractory and radioiodine-responsive in patients with DTC. ROC = receiver operating characteristic, TERT = telomerase reverse transcriptase.

**Figure 2.**
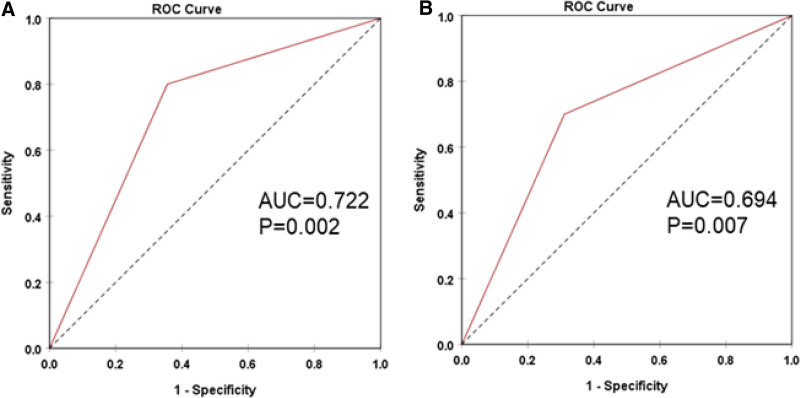
Prognostic performance of *BRAF V600E* and *TERT* promoter mutations for postoperative recurrence in patients with RAIR-DTC. ROC curve of *TERT* promoter mutation and *BRAF* V600E (A) of and *TERT* promoter co-mutation (B) in differentiating recurrence and non-recurrence after surgery in patients with RAIR-DTC. RAIR-DTC = radioiodine-refractory differentiated thyroid cancer, ROC = receiver operating characteristic, TERT = telomerase reverse transcriptase.

**Figure 3.**
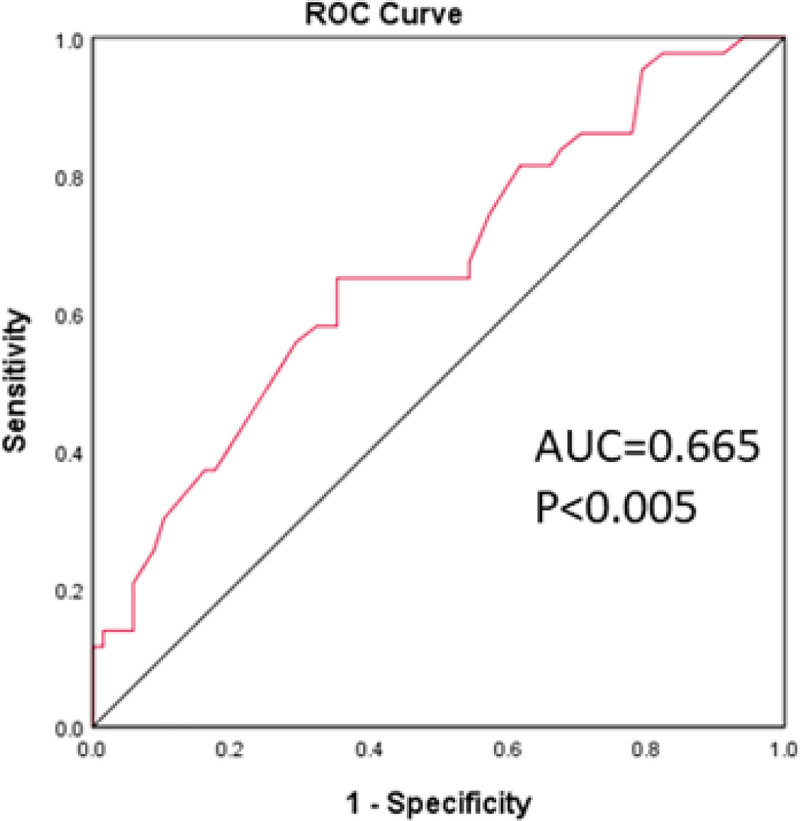
ROC curve model of age predicting the co-mutation status of *BRAF V600E* and *TERT* promoter in patients with RAIR-DTC. RAIR-DTC = radioiodine-refractory differentiated thyroid cancer, ROC = receiver operating characteristic, TERT = telomerase reverse transcriptase.

We analyzed the prognostic performance of the *BRAF V600E* and *TERT* promoter mutations for loss of radioiodine avidity in patients with DTC and observed that *TERT* promoter mutation can discriminate radioiodine-refractory and radioiodine-responsive (AUC = 0.63) (sensitivity of 44.1% and specificity of 81.8%) (Fig. 1). These results indicated that *TERT* promoter mutation could be an additional biomarker for the prognosis of loss of radioiodine avidity in patients with DTC. Both mutations showed moderate predictive value for recurrence, with the *TERT* promoter mutation (AUC = 0.72) (sensitivity of 80% and specificity of 64.4%) and the co-mutation (AUC = 0.69) (sensitivity of 70% and specificity of 68.9%) (Fig. 2). This suggests that these mutations may be potential biomarkers for predicting recurrence in RAIR-DTC patients. Furthermore, ages were also identified as a predictive factor for the presence of *BRAF V600E* and *TERT* promoter co-mutations in patients with RAIR-DTC (AUC = 0.67), with a cutoff value of 46 years (sensitivity of 65.1% and specificity of 64.7%) (Fig. 3).

### 3.5. Recurrence-free survival (RFS) of patients with DTC

**Figure 4.**
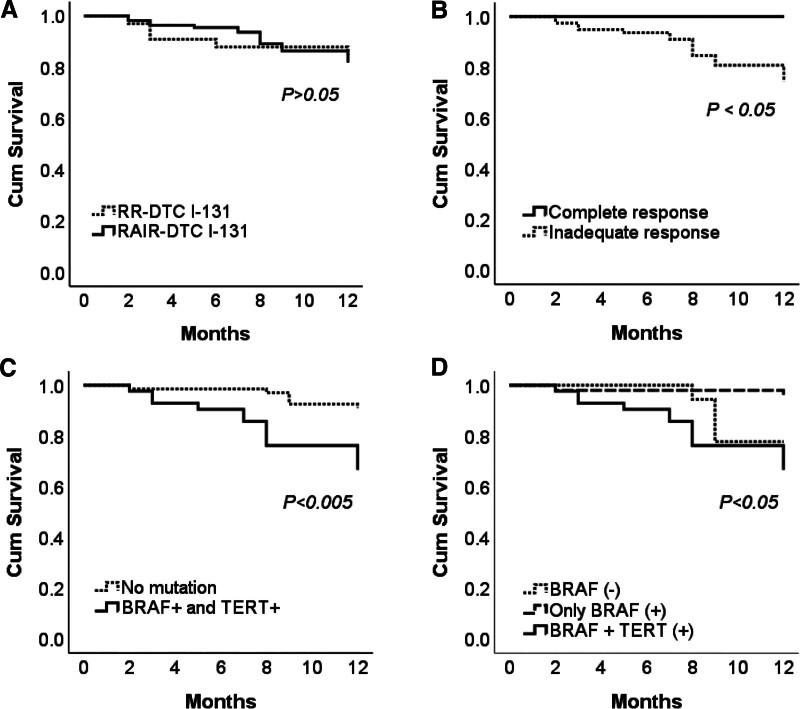
Kaplan–Meier plot comparing progression-free survival after surgery for recurrence. (A) Between radioiodine-refractory differentiated thyroid cancer (RAIR-DTC) and radioiodine-responsive differentiated thyroid cancer (RR-DTC); (B) between complete and inadequate responses groups. (C) Between patients with both *BRAF* and *TERT* mutations versus those without *BRAF* and *TERT* mutations. (D) Among patients with both *BRAF* and *TERT* mutations, those with only *BRAF* mutation and those without *BRAF* mutation. *P* values were calculated by the Mantel-Cox log-rank test. TERT = telomerase reverse transcriptase.

We conducted a RFS analysis and found that the 1-year progression-free survival rate after reoperation for recurrence in the RAIR-DTC group was 81.8%, lower than the 84.8% observed in the RR-DTC group. However, the difference between the 2 groups was not statistically significant (*P* > .05). The postoperative RFS in RAIR-DTC patients with *BRAF V600E* and *TERT* promoter co-mutations (10.55 months) was significantly lower than in those without the co-mutation (11.66 months) (*P* = .001). These findings support the hypothesis that the coexistence of *BRAF V600E* and *TERT* promoter mutations is a poor prognostic factor, increasing the risk of recurrence. Additionally, we observed that the RFS in the coexistence of *TERT* promoter and *BRAF V600E* mutations (10.5 months) was shorter than in the *BRAF V600E* negative group (11.3 months) and the BRAF mutation only group (11.8 months) (*P* < .05). This suggests that the *TERT* promoter mutation plays a crucial role in disease progression when coexisting with *BRAF V600E* (Fig. 4). Furthermore, we evaluated the impact of treatment responses on RFS in RAIR-DTC patients. Our results indicate that patients with incomplete treatment responses had significantly shorter postoperative RFS (*P* < .05) (Fig. 4). These findings suggest that these incomplete treatment responses are poor prognostic factors for RFS in RAIR-DTC patients.

## 4. Discussion

The global incidence of thyroid cancer has been rising in recent years.^[22]^ Advances in diagnosis and treatment have led to a generally favorable prognosis, with a 10-year survival rate of up to 93% for PTC patients undergoing surgery and ¹³¹I therapy. However, managing RAIR-DTC remains a major challenge, as studies estimate a median survival of only 2.5 to 3.5 years for patients with distant metastases.^[4]^ Understanding the mechanisms of radioiodine resistance and identifying predictive biomarkers are crucial for improving treatment strategies. Mutations such as *BRAF V600E* and *TERT* promoter are believed to drive MAPK/ERK and PI3K/AKT pathway activation, contributing to radioiodine resistance.^[23]^ In this study, we report a high frequency of *BRAF V600E* and *TERT* promoter mutations in Vietnamese DTC patients, their associations with clinical parameters, and their prognostic significance.

A meta-analysis has shown that *TERT* promoter mutations are associated with various factors, including sex, age, tumor size, vascular invasion, extrathyroidal extension, lymph node and distant metastases, advanced TNM stage, recurrence, and disease-specific mortality in DTC and PTC patients.^[24]^ In our study, *TERT* promoter mutations were detected in 38.2% of cases, a significantly higher prevalence than the 3.4% reported in a previous study on thyroid carcinoma.^[25]^ Our findings indicate that DTC patients with *TERT* promoter mutations have a 2.55-fold increased risk of radioiodine resistance compared to those without this mutation. This aligns with Liu et al, who reported that recurrent PTC patients with *TERT* promoter mutations were 6.89 times more likely to lose radioiodine avidity than those with the wild-type phenotype.^[18]^ Similarly, Yang et al found a significant association between *TERT* mutations and poor response to radioiodine therapy.^[26]^ These results suggest that *TERT* mutations may serve as a molecular marker for radioiodine resistance in DTC patients. In contrast, we found no association between the *BRAF V600E* mutation and loss of radioiodine avidity, consistent with earlier study reported no significant link between *BRAF* mutations and clinical response to radioactive iodine therapy in PTC patients without distant metastases.^[27]^ However, another study did find an association between *BRAF* mutations and loss of radioiodine avidity.^[18]^ Variations in findings across studies may arise from differences in study design, clinical context, and underlying genetic backgrounds. Thus, further investigations in independent and ethnically diverse cohorts are warranted to better elucidate the impact of *BRAF V600E* mutations on radioiodine uptake in DTC patients.

Studies suggest that the coexistence of *TERT* promoter and *BRAF V600E* mutations may synergistically drive tumorigenesis.^[28,29]^ Patients with recurrent PTC harboring both *BRAF* and *TERT* mutations face a higher risk of losing radioiodine avidity compared to those with either mutation alone.^[18]^ However, our study found no significant difference in the risk of radioiodine resistance between DTC patients with both *BRAF V600E* and *TERT* mutations and those without these mutations. Additionally, repeated radioiodine treatment may induce adaptive resistance in thyroid cancer cells. Our findings revealed a significant association between the total cumulative dose of radioiodine and resistance in DTC patients. This highlights the importance of early identification of RAIR-DTC to prevent unnecessary repeat treatments and optimize patient management.

The *TERT* promoter mutation in thyroid cancer is linked to more aggressive clinical outcomes. In this study, we observed an association between *TERT* promoter mutations and increased primary tumor size in RAIR-DTC patients. Liu et al reported that DTC patients with the *TERT* promoter mutation had larger tumors compared to those without this mutation,^[17]^ reinforcing its role in thyroid cancer progression and its potential as a predictor of clinical outcomes in thyroid cancer patients. Our findings highlight *TERT* promoter mutations and *BRAF V600E* + *TERT* co-mutations as significant prognostic factors in RAIR-DTC, predicting radioiodine resistance, recurrence, and shorter RFS. *TERT* mutations demonstrated high sensitivity for recurrence (80%), while co-mutations had a strong impact on survival, consistent with previous studies.^[17,30]^ Xing et al reported median RFS of approximately 2 to 3 years in co-mutated DTC patients, whereas RAIR-DTC patients showed significantly shorter survival (10–11 months), reflecting their refractory nature.^[30]^ The synergy between *TERT* and *BRAF* likely accelerates dedifferentiation and radioiodine resistance.^[31]^ A recent study has elucidated how *TERT* drives BRAF mutant-induced thyroid cancer dedifferentiation and progression by enhancing ribosomal biogenesis, a key process supporting cancer cell survival, proliferation, differentiation, and metastasis.^[32]^ These insights underscore the need for personalized treatment strategies, closer monitoring of *TERT*-positive patients, and alternative therapies for those with co-mutations.

Although our findings confirm the impact of *TERT* promoter and *BRAF V600E* mutations in DTC and highlight their prognostic value in predicting radioiodine resistance and recurrence, the study has several limitations. First, the small number of RR-DTC patients may limit statistical significance. Second, the 1-year follow-up period may be insufficient to fully assess the long-term effects of these mutations on DTC progression. Third, the moderate predictive power of AUC values for mutation-based predictions may be another limitation in using mutation-based markers alone for prognostication, thus, they should be interpreted in conjunction with other clinical and pathological features to enhance predictive accuracy. Future studies should validate these findings in larger, long-term, independent and/or external cohorts as well as explore additional combinatorial biomarkers.

In conclusion, our study indicates that *TERT* promoter mutations significantly increase the risk of RAI treatment failure in DTC patients. The coexistence of *TERT* promoter and *BRAF V600E* mutations further elevates the likelihood of losing radioiodine avidity. These mutations may serve as an early biomarker for predicting radioiodine resistance. Additionally, careful management of RAI treatment doses is crucial to reducing the risk of resistance and optimizing therapeutic outcomes in DTC patients.

## Acknowledgments

We thank all patients for their participation.

## Author contributions

**Conceptualization:** Le Ngoc Ha, Hoang Van Tong.

**Data curation:** Le Thi Tuyet Nhung, Nghiem Xuan Hoan, Dao Phuong Giang, Dang Trung Dung, Nguyen Thi Phuong, Ngo Thi Minh Hanh, Ngo Thu Hang.

**Formal analysis:** Le Van Khanh, Hoang Van Tong.

**Investigation:** Le Thi Tuyet Nhung, Nghiem Xuan Hoan, Dao Phuong Giang.

**Methodology:** Nghiem Xuan Hoan, Dao Phuong Giang, Le Ngoc Ha.

**Resources:** Nghiem Xuan Hoan, Le Ngoc Ha.

**Supervision:** Le Ngoc Ha, Hoang Van Tong.

**Validation:** Le Ngoc Ha, Hoang Van Tong.

**Visualization:** Le Thi Tuyet Nhung, Nghiem Xuan Hoan, Ngo Thu Hang.

**Writing – review & editing:** Le Thi Tuyet Nhung, Nghiem Xuan Hoan, Hoang Van Tong.

**Writing – original draft:** Le Van Khanh, Hoang Van Tong.
